# Comparative Efficacy and Safety of Nivolumab and Nivolumab Plus Ipilimumab in Advanced Cancer: A Systematic Review and Meta-Analysis

**DOI:** 10.3389/fphar.2020.00040

**Published:** 2020-02-14

**Authors:** Yi Yang, Gang Jin, Yao Pang, Yijie Huang, Wenhao Wang, Hongyi Zhang, Guangxin Tuo, Peng Wu, Zequan Wang, Zijiang Zhu

**Affiliations:** ^1^ Department of Thoracic Surgery, Gansu Provincial Hospital, Lanzhou, China; ^2^ Department of Clinical Medicine, Gansu University of Traditional Chinese Medicine, Lanzhou, China; ^3^ School of Health Preservation and Rehabilitation, Chengdu University of Traditional Chinese Medicine, Chengdu, China

**Keywords:** nivolumab, ipilimumab, advanced cancer, combination immunotherapy, efficacy, safety

## Abstract

**Background:**

Combination therapy with immune checkpoint inhibitors (ICIs) has been applied in the clinic to achieve synergistic effects and to improve clinical efficacy. Compared with monotherapy, combination therapy has promising efficacy against various advanced cancers. To further verify the effectiveness of combination therapy, we conducted a meta-analysis of the efficacy and safety of nivolumab (NIVO) and NIVO plus ipilimumab (IPI) in advanced cancer.

**Methods:**

Electronic databases (PubMed, EMbase, and The Cochrane Library) were systematically searched for applicable studies published in English between January 1990 and June 2019. Relevant outcomes included objective response rate (ORR), disease control rate (DCR), median progression-free survival (mPFS), median overall survival (mOS), and grade 3–4 adverse events (AEs).

**Results:**

A total of 1,297 patients from six studies were included. Compared with NIVO alone, NIVO + IPI was more efficacious for advanced tumors. Pooled outcome values were: ORR, 1.73 (95% CI: 1.34–2.23); DCR, 1.80 (95% CI: 1.21–2.69); mPFS, 0.22 (95% CI: 0.03–0.41); mOS, 0.03 (95% CI: −0.20–0.26); and grade 3–4 AEs, 3.64 (95% CI: 2.86–4.62).

**Conclusion:**

NIVO + IPI is more effective than NIVO alone for the treatment of advanced cancer and can significantly improve ORR and DCR and prolong mPFS. Due to the limited quality and quantity of the included studies, more high-quality studies are needed to validate the above conclusions.

## Introduction

Cancers remain difficult to cure because the inherent intrinsic genomic instability of tumors facilitates their escape from cytotoxicity and targeted therapy (Miller and Sadelain, 2015). However, the discovery of cancer immune checkpoints and the success of immune checkpoint inhibitors (ICIs) may improve patient survival.

Cytotoxic T-lymphocyte-associated antigen-4 (CTLA-4) (Pardoll, 2012), programmed cell death-1 and its ligands (PD-1/PD-L1/2) (Topalian et al., 2012), and lymphocyte activation gene-3 (Yu et al., 2019) inhibit the T cell immune response. CTLA-4 signaling limits the initiation of the T cell response in the lymph nodes early in the immune response, whereas PD-1 restricts T cell activity later in the process in the tumor microenvironment (Fife and Bluestone, 2008). The CTLA-4 and PD-1–PD-L1/PD-L2 checkpoints are commonly exploited by tumors to evade and/or suppress the immune system. Therefore, many monoclonal antibodies have been developed to block proteins that are involved in the downregulation of immune responses (Meng et al., 2015; Ngiow et al., 2015) by stimulating T cell-dependent cytotoxicity against tumor cells through abrogating peripheral tolerance (Cuende et al., 2015). Therefore, the use of monoclonal antibodies to block immune checkpoints has become a promising cancer treatment strategy (Anagnostou et al., 2017) and can lead to long-lasting antitumor activity, improving survival rates for various malignancies compared with other systemic therapies (Bang et al., 2017). Anti-CTLA-4 antibody (ipilimumab [IPI]), anti-PD-1 antibodies (nivolumab [NIVO] and pembrolizumab), and anti-PD-L1 antibodies (atezolizumab, avelumab, and durvalumab) have been approved for clinical use in various advanced solid tumors, such as melanoma (Hodi et al., 2016), nonsmall cell lung cancer (NSCLC) (Dal Bello et al., 2017), renal cell cancer (Atkins et al., 2017), small cell lung cancer (Schneider and Kalemkerian, 2016), gastro-esophageal cancer, and liver cancer (Gong et al., 2018).

NIVO is a human IgG4 PD-1 ICI antibody that selectively blocks the PD-1 receptor on the surface of cytotoxic T cells to prevent downregulation of the immune response in malignant tumor cells induced by PD-L1 (Minguet et al., 2016). Because it has been shown to significantly improve overall survival (OS) and safety in selected patients, NIVO has been approved by the United States (US) and the European Union (EU) for the treatment of locally advanced or metastatic NSCLC (Minguet et al., 2016; Vokes et al., 2018), advanced renal cell carcinoma, and advanced melanoma (Larkin et al., 2015; Raedler, 2015; Wolchok et al., 2017; Schuyler, 2018). In addition, NIVO can treat recurrent or refractory Hodgkin's lymphoma with good efficacy and safety (Ansell et al., 2015). Ipilimumab (IPI) is a human monoclonal IgG4 that acts as an antineoplastic ICI by selectively binding to cytotoxic T lymphocyte-associated antigen 4, a molecule located on the surface of cytotoxic T cells, suppressing the immune response (Hodi et al., 2010). IPI blocks CTLA-4, leading to a continuously active immune response in malignant cells. The US and EU have approved IPI monotherapy to treat melanoma (Lipson and Drake, 2011).

Although significant progress has been made, the effect of immunotherapy is not completely satisfactory. Despite some durable responses, most patients did not respond to their initial treatment (primary resistance) and some responders later relapsed (acquired resistance). Insufficient infiltration of cytotoxic T lymphocytes, lack of tumor-associated antigens, or activation of other immunosuppressive pathways are significant causes of resistance to immunotherapy (Sharpe and Pauken, 2018).

Compared with monotherapy, ICI-combined therapy can provide a significant OS benefit. Combination therapy has been shown to be efficacious against different malignancies; clinical data show that chemotherapy can induce the expression of PD-L1 in tumor cells and regulate their immune function (Wei et al., 2019). The combination of anti-CTLA4 and anti-PD1 leads to significantly better response rates and progression-free survival than anti-PD1 agents alone. In patients with metastatic melanoma, NIVO monotherapy and NIVO + IPI treatment resulted in significantly longer median progression-free survival (PFS) than chemotherapy or IPI treatment (Hodi et al., 2016; Hao et al., 2017). The mechanism might involve enhanced simultaneous blockade of the CTLA-4 and PD-1 pathways, cell infiltration, and/or activated expression of markers and inflammatory cytokines (Curran et al., 2010). Additionally, a greater ratio of CD8^+^ T cells to regulatory T cells and myeloid-derived suppressor cells in the tumor may contribute to multiple coinhibitory blockades. However, combination therapy might increase the incidence of adverse events (AEs). The vast majority of these are grade 3–4 AEs that appear in the first few weeks to months after treatment initiation, and the most common ones include pruritus, nausea, rash, diarrhea, and atony. There are also some serious grade 5 AEs, such as pneumonia, neurotoxic effects, myocarditis, and hepatitis, some of which may be fatal (Omuro et al., 2018). The efficacy and safety of combination immunotherapy is still controversial, thus we undertook the current meta-analysis.

## Materials and Methods

The current systematic review and meta-analysis conformed with the Preferred Reporting Items for Systematic Reviews and Meta-Analyses (PRISMA) statement.

### Search Strategy

We searched PubMed, Embase, and The Cochrane Library databases for relevant English-language articles that had been published by 1 June 2019. The following terms were used: (nivolumab or Opdivo) AND (ipilimumab or Yervoy) AND (neoplasm* OR tumor* OR cancer* OR malignant* OR malignant neoplasm*). We also performed a manual search to find applicable studies in the references and related citations.

### Eligibility Criteria

We included studies that fulfilled the following criteria: (a) population, patients with stage III–IV malignancies; (b) intervention, NIVO + IPI; (c) control, NIVO monotherapy; (d) prospective study, phase II or III clinical trials; and (e) inclusion of any of the outcome measures. Where multiple articles had analyzed the same trial, the most recent study was used.

### Outcome Measures

The primary outcomes were objective response rate (ORR, percentage of patients who achieved an objective response as defined by the Response Evaluation Criteria in Solid Tumors), disease control rate (DCR), mPFS, median OS (mOS), and AEs. The severity of AEs was graded according to the National Cancer Institute Common Terminology Criteria for Adverse Events, version 4.0.

### Data Extraction

Two reviewers independently screened the titles and abstracts of retrieved citations. Discrepancies were resolved by discussion. A standardized extraction form was prepared using Microsoft Excel (Microsoft, Redmond, Washington). The extracted data included first author, study design, population, information for assessment for risk of bias (ROB), treatments, and measured outcomes (ORR, DCR, mPFS, mOS, grade 3–4 AEs).

### ROB Assessment

ROB was assessed by two independent reviewers using the Cochrane Collaboration's tool for ROB assessment (Lundh and Gotzsche, 2008).

### Statistical Analysis

For the meta-analysis, we estimated the standard mean difference for continuous outcomes. Odds ratio (OR) was used to compare dichotomous variables, and Peto odds ratio was used to compare rare AEs. All the results were reported with 95% confidence intervals (CI). Pooled OR and 95% CIs for dichotomous data were estimated using the Mantel–Haenszel method. The *I*-square (*I* ^2^) test was performed to assess the impact of study heterogeneity. If severe heterogeneity was present at *I* ^2^ > 50%, the random effect model was chosen; otherwise, the fixed-effect model was used. In the case of a missing SD of the mean change from baseline, it was calculated from the SE or the 95% CI. We used Review Manager (RevMan, version 5.3; Copenhagen: The Nordic Cochrane Centre, The Cochrane Collaboration, 2014).

## Results

### Search Results and Studied Characteristics

**Figure 1:**
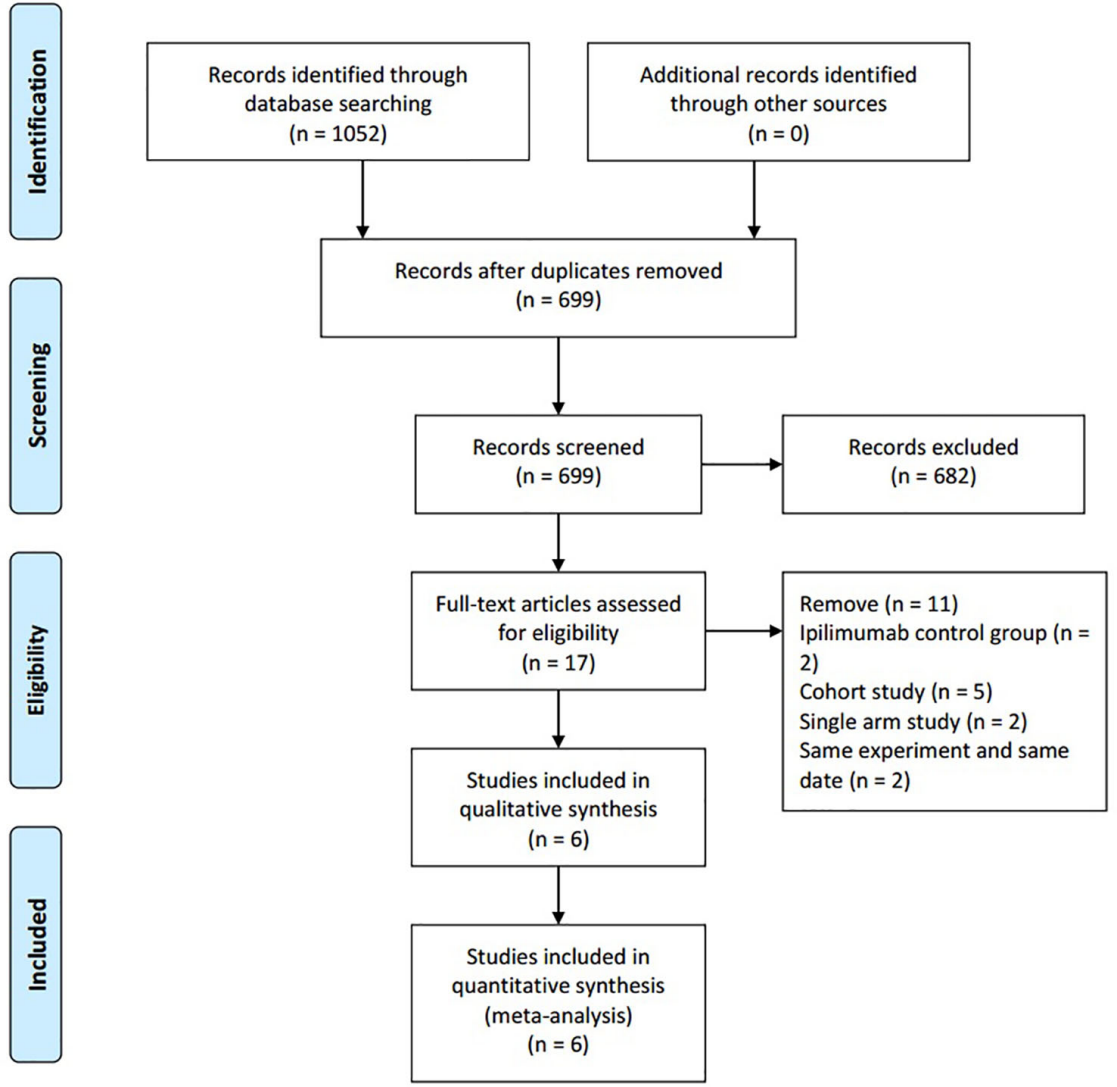
PRISMA chart.

The initial search identified 1,052 publications. After excluding duplicates, 699 publications remained. Of these, 682 studies were discarded after reading the titles and abstracts. After assessing the full texts, 11 reports were further excluded and six studies were included for data analysis. Details regarding the selection of studies are outlined in the flow diagram in **Figure 1**. The included studies were published between 2018 and 2019. The six studies were all randomized controlled trials (D'Angelo et al., 2018; Hodi et al., 2018; Janjigian et al., 2018; Long et al., 2018; Scherpereel et al., 2019; Sharma et al., 2019) and included 1,189 patients with advanced-stage cancers. There were five phase II studies and one phase III study. The intervention group received intravenous NIVO (3 mg/kg) + IPI (1 mg/kg) or intravenous NIVO (1 mg/kg) + IPI (3 mg/kg), while the control group received intravenous NIVO (3 mg/kg) (**Table 1**).

**Table 1:** Characteristics of included studies.

No. Study	Trial phase	Study design	Disease	Participants	Intervention	Comparator	No. of patients (I/C)	Ages(years)		ECOG	
								I	C	I	C
Scherpereel et al., 2019	II	Multicenter open-label, randomized noncomparative,	relapsed malignant pleural mesothelioma	Patients were aged 18 years or older, histologically proven malignant pleural mesothelioma progressing after first-line or second-line pemetrexed and platinum based treatments, measurable disease by CT,	NIVO (3 mg/kg every 2 weeks) + IPI (1 mg/kg every 6 weeks)	NIVO (3 mg/kg) every 2 weeks	125 (62/63)	71.2 (48.1–88.1)	72.3 (32.5–87.2)	0:25 1:36 2:1	0:19 1:42 2:0
Sharma et al., 2019	I/II	Multicenter open-label multiarm randomly assigned	Metastatic Urothelial Carcinoma	Patients in the locally advanced or metastatic platinum pretreated urothelial carcinoma	NIVO 3 mg/kg + IPI 1 mg/kg every 3 weeks for four doses	NIVO (3 mg/kg) every 2 weeks	182 (104/78)	63.0 (39–83)	65.5 (31–85)	0:40 1:64	0:42 1:36
D'Angelo et al., 2018	II	two open-label, noncomparative, randomized,	metastatic sarcoma	patients aged 18 years or older and had central pathology confirmation of sarcoma with at least one measurable lesion, evidence of metastatic, locally advanced or unresectable disease,	NIVO 1 mg/kg + IPI 3 mg/kg every 3 weeks for four doses	NIVO (3 mg/kg) every 2 weeks	85 (42/43)	57.0 (27.0–81.0)	56.0 (21.0–76.0)	0:24 1:18	0:28 1:15
Hodi et al., 2018	III	multicenter, randomized	advanced melanoma	Patients were aged 18 years or older with previously untreated, unresectable, stage III or stage IV melanoma, known BRAFV600 mutation status.	NIVO 1 mg/kg + IPI 3 mg/kg every 3 weeks for four doses	NIVO (3 mg/kg) every 2 weeks	630 (314/316)	–	–	–	–
Janjigian et al., 2018	III	open-label two-stage randomized	Metastatic Esophagogastric	Patients with locally advanced or metastatic chemotherapy–refractory gastric, esophageal, or gastroesophageal junction cancer from centers in the United States and Europe	NIVO 1 mg/kg + IPI 3 mg/kg every 3 weeks for four doses	NIVO (3 mg/kg) every 2 weeks	108 (49/59)	53 (27–77)	60 (29–80)	0:27 1:22	0:29 1:30
Long et al., 2018	II	multicenter randomized	melanoma brain metastases	Immunotherapy-naive patients aged 18 years or older with melanoma brain metastases.	NIVO 1 mg/kg + IPI 3 mg/kg every 3 weeks for four doses	NIVO (3 mg/kg) every 2 weeks	63 (35/25)	59 (53–68)	63 (52–74)	0+1:34 2:1	0+1:25

### Quality Assessment

**Figure 2:**
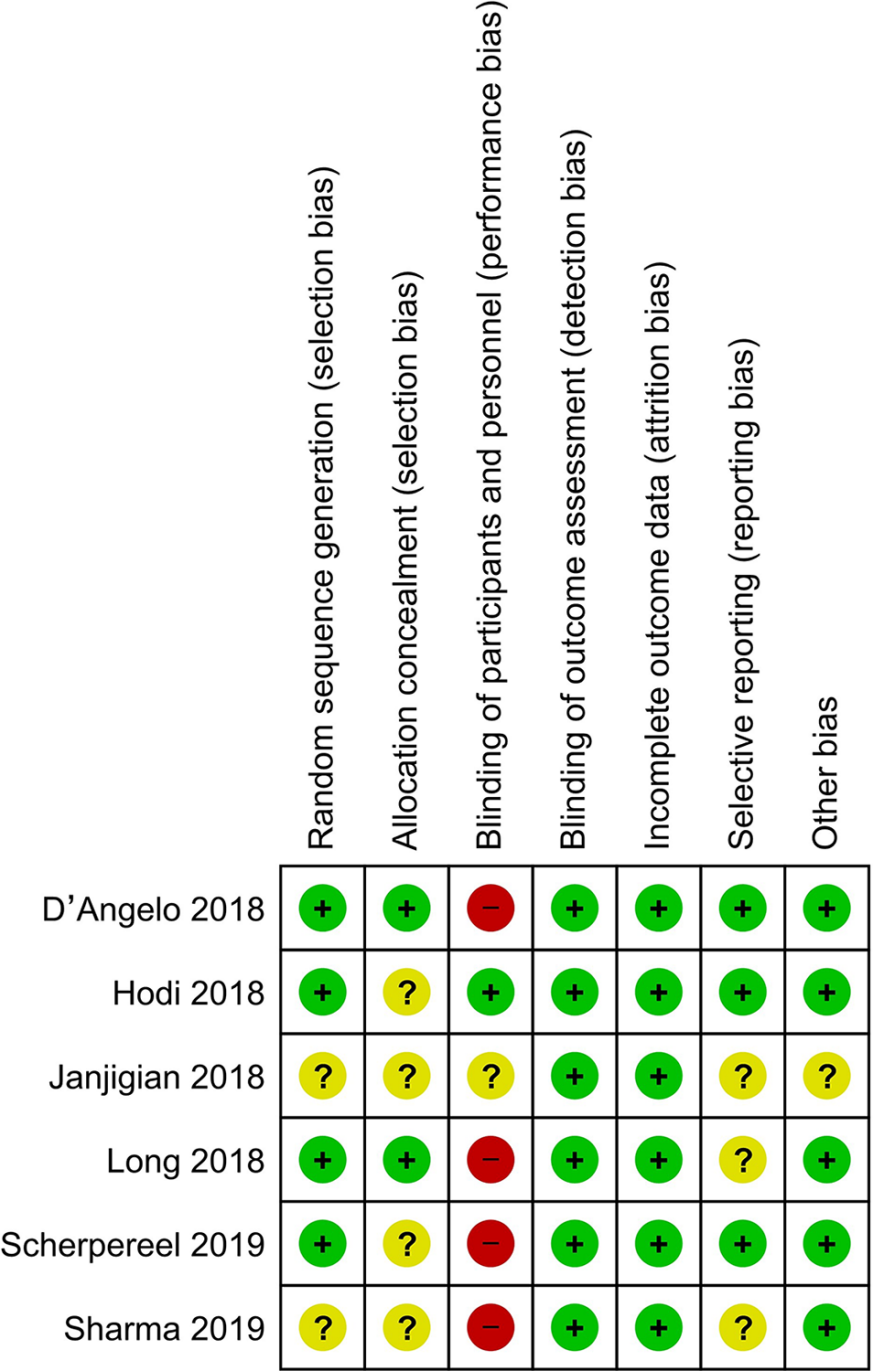
Risk of bias of included studies.

The results of the quality assessment are shown in **Figure 2**. Most studies had a low risk of bias. Random sequence generation was not found in two studies (Janjigian et al., 2018; Scherpereel et al., 2019), and some studies did not clearly report concealment (Janjigian et al., 2018; Hodi et al., 2018; Scherpereel et al., 2019; Sharma et al., 2019). The blinding of participants was explicitly reported in only one study (Hodi et al., 2018). Furthermore, some studies did not clearly report selective reporting (Long et al., 2018; Sharma et al., 2019; Janjigian et al., 2018) or other bias (Janjigian et al., 2018).

### Efficacy

**Figure 3:**
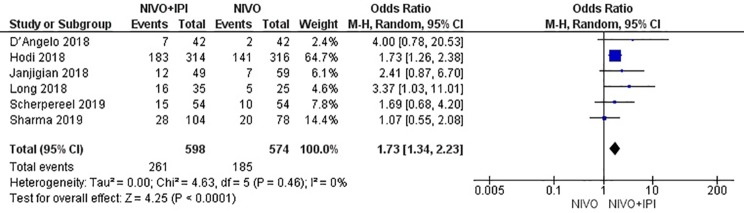
Forest plot of nivolumab plus ipilimumab *vs*. nivolumab for improving ORR.

**Figure 4:**
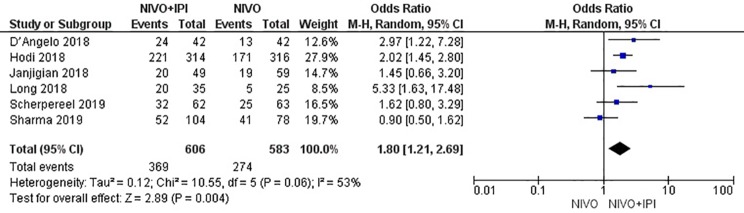
Forest plot of nivolumab plus ipilimumab *vs*. nivolumab for improving DCR.

**Figure 5:**
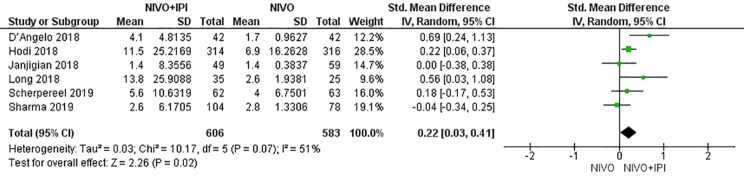
Forest plot of nivolumab plus ipilimumab *vs*. nivolumab for prolonging mPFS.

**Figure 6:**
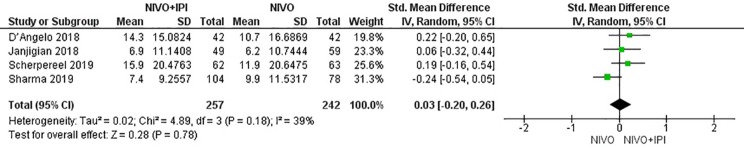
Forest plot of nivolumab plus ipilimumab *vs*. nivolumab for prolonging mOS.

The efficacy of NIVO + IPI or NIVO for advanced tumors was evaluated by combining ORR, DCR, mPFS, and mOS. We included all six studies to analyze ORR, DCR, and mPFS, and four studies to evaluate mOS. The combined results revealed an ORR of 1.73 (95% CI: 1.34–2.23, *I* *^2^* = 0%, *P* = 0.46), suggesting that compared with NIVO monotherapy, patients were more likely to respond to NIVO + IPI therapy, thus improving the ORR. The DCR was 1.80 (95% CI: 1.21–2.69, *I^2^* = 53%, *P* = 0.06), showing that the PFS of the NIVO + IPI group could control the progression of cancer better than the NIVO group. There was heterogeneity between these two studies and the random effect model was used. PFS was 0.22 (95% CI: 0.03–0.41, *I^2^* = 51%, *P* = 0.07), indicating that the PFS of the NIVO + IPI group was significantly improved when compared with the NIVO group. There was slight heterogeneity among the studies and the random effect model was used. OS was 0.03 (95% CI: ^−^0.20–0.26, *I^2^ =* 39%, P = 0.18), and there was no statistical difference between the NIVO + IPI group and the NIVO group. Significant differences in ORR, DCR, and mPFS were found. These results are shown in **Figures 3**–**6**.

### Safety

**Figure 7:**
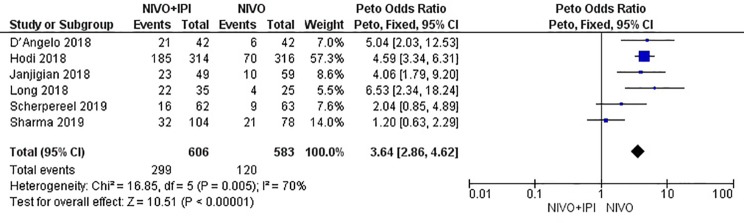
Forest plot of nivolumab plus ipilimumab *vs*. nivolumab for increasing grade 3–4 AEs.

The combined incidence of grade 3–4 AEs in the six included studies was 3.64 (95% CI: 2.86–4.62; *I^2^* = 70%; *P* = 0.005); the results showed that the incidence of AEs in the NIVO + IPI group was higher than that in the NIVO group. The total risk of AEs significantly differed between the combination and monotherapy arms (**Figure 7**). The most common AEs in the combined treatment group (n = 606) were hepatotoxicity (n = 71, 11.71%), diarrhea (n = 49, 8.08%), increased lipase (n = 44, 7.26%), rash (n = 27, 4.45%), and fatigue (n = 24, 3.96%). The most common AEs in the monotherapy group (n = 583) were increased lipase (n = 26, 4.45%), hepatotoxicity (n = 13, 2.22%), diarrhea (n = 11, 1.88%), rash (n = 10, 1.71%), and fatigue (n = 9, 1.54%).

### Publication Bias Test and Sensitivity Analysis

Publication bias analysis was not performed because our analysis included fewer than 10 studies. Sensitivity analysis was performed on the results, but no significant change was observed after the fixed effect model was adopted, indicating that the results of this study were stable (**Table 2**).

**Table 2:** Sensitivity analysis.

	objective response rate (ORR)	disease control rate (DCR)	median progression-free survival (mPFS)	median overall survival (mOS)
Random Effect Model	1.73 (95% CI: 1.34–2.22)	1.79 (95% CI: 1.18–2.72)	0.22 (95% CI: 0.03–0.41)	0.03 (95% CI: −0.20–0.26)
Fixed Effect Model	1.73 (95% CI: 1.35, 2.23)	1.78 (95% CI: 1.41, 2.25)	0.20 (95% CI: 0.09, 0.32)	0.01 (95% CI: −0.16, 0.19)

## Discussion

Our meta-analysis showed that NIVO + IPI combined immunotherapy significantly improved antitumor efficacy and led to better ORR and DCR compared with NIVO monotherapy. Combined treatment was also associated with longer PFS, but OS did not significantly differ between the two groups. Adverse events ≥ grade 3 were more frequent but controllable in the combined treatment arm.

In the meta-analysis, we found that combination therapy was superior to monotherapy. This may be because: (a) the efficacy of monotherapy is limited by low response rates, with only a small proportion of patients responding to treatment (Rotte et al., 2018; Hellman et al., 2018) (b) combining anti-CTLA-4 and anti-PD-1 therapies was suggested to activate the antitumor immune response synergistically, thus increasing response rates (Curran et al., 2010); (c) combining anti-CTLA-4 and anti-PD-1 therapies significantly increases the ratios of both CD8^+^/regulatory T cells and CD4^+^ effector/regulatory T cells within the tumor, so that CD8^+^ and CD4^+^ T cells to continue to survive, proliferate, and perform effector functions in the tumor (Duraiswamy et al., 2013; Beavis et al., 2018); (d) combining anti-CTLA-4 and anti-PD-1 therapies allows the accumulation of active T cells that express CTLA-4 and PD-1 and would otherwise be energized (Curran et al., 2010); and (e) combining anti-CTLA-4 and anti-PD-1 therapies increases the production of inflammatory cytokines (such as IFN-*γ* and TNF-*α*) in the tumor itself and in its draining lymph nodes (Shi et al., 2016). Some clinical trials support this idea. Combined immunological checkpoint blockade synergistically inhibited tumor immune escape, and thus improved the efficacy of single-agent anti-PD-1 therapy in esophagogastric cancer; however, the clinical effect was not related to the expression of tumor PD-L1 (Janjigian et al., 2018). A previous study (Hodi et al., 2018) reported that NIVO + IPI or NIVO monotherapy could achieve lasting and sustained clinical efficacy in patients with advanced melanoma regardless of *BRAF* mutation status. Although the efficacy of NIVO monotherapy was better supported, combination therapy was more likely to prolong survival than NIVO monotherapy. However, PD-L1 levels did not predict the efficacy of combination therapy. Similar to Hodi's research, NIVO + IPI was a suitable first-line treatment for asymptomatic brain metastases, and patients whose baseline biopsy PD-L1 expression was ≥1% had a numerically higher overall mPFS than did patients whose tumor PD-L1 expression was <1% (Long et al., 2018). Other studies (Scherpereel et al., 2019) have pointed out that the combined regimen was most effective in patients with PD-L1^+^ malignant pleural mesothelioma, especially in patients whose tumors had high PD-L1 expression (≥25% positive cells). This view was also supported by a single-arm experiment (Disselhorst et al., 2019). A recent study (D'Angelo et al., 2018) reported that patients with locally advanced, unresectable, or metastatic soft-tissue sarcomas who received combination immunotherapy achieved significant therapeutic effects compared with patients who received monotherapy, but this study did not mention biomarkers that could predict prognosis. Identifying highly sensitive and specific immunotherapeutic biomarkers is an important topic in oncology. In contrast, monotherapy has been shown to be superior to combination therapy for glioblastoma (Omuro et al., 2018). The lesser efficacy in the combination group might reflect ICI-enhanced inflammatory infiltration in some patients with central nervous system tumors. Of note, based on previous research, the survival benefit for patients whose tumors have >1% PD-L1+ cells is greater than for patients whose tumors have <1% PD-L1+ cells (Brahmer et al., 2012). However, some of our included studies found that the therapeutic effect was unrelated to PD-L1 expression. Tumor mutation burden (TMB) has shown some clinical predictive value in clinical trials^[33]^.

A recent study also found that the effects in the combined group were influenced by the doses of both drugs (Rozeman et al., 2019); they identified a tolerable combination dose plan (two cycles of NIVO 1 mg/kg + IPI 3 mg/kg) with a high response rate. Another study compared different doses (Sharma et al., 2019) and found that the effective rates of NIVO (3 mg/kg) + IPI (1 mg/kg) *versus* NIVO (1 mg/kg) + IPI (3 mg/kg) were 26.9% and 38.0%, respectively, and mPFS was 2.6 months (95% CI: 1.4–3.9) *versus* 4.9 months (95% CI: 2.7–6.6). Administration cycles also affected outcomes: mPFS was 8.1 months (95% CI: 5.6–13.6) or 3.9 months (95% CI: 2.6–13.2) using NIVO (3 mg/kg) + IPI (1 mg/kg) every 12 or 6 weeks, respectively. Twelve-week cycles appear to be safe.

Of note, NIVO + IPI combination immunotherapy was shown to be effective in many clinical trials that did not meet our study inclusion criteria. NIVO + IPI showed significant advantages over sunitinib in advanced renal cell carcinoma (Motzer et al., 2018), which led to FDA approval of NIVO + IPI for the treatment of advanced renal cell carcinoma (Schuyler, 2018; Cella et al., 2019). Another study (Reck et al., 2019) showed that first-line NIVO + IPI led to continuous early improvement in patients with advanced NSCLC and high TMB compared with chemotherapy. Japan's single-arm experiment (Namikawa et al., 2018) also highlighted the advantages of NIVO + IPI.

This meta-analysis also evaluated grade 3–4 AEs. The combined treatment groups in our study had a higher overall incidence of AEs than the monotherapy groups. The most common AEs associated with combined immunotherapy were hepatotoxicity, diarrhea, increased lipase, fatigue, and rash. Therefore, preventing or treating these AEs among patients who receive these combinations should be considered. Four deaths that might have been associated with combination therapy were reported, including one each from tumor lysis syndrome (Sharma et al., 2019), fulminant hepatitis, encephalitis, and acute kidney failure (Scherpereel et al., 2019).

Other studies analyzed the potential causes of toxicity (Sharma et al., 2019). The NIVO (1 mg/kg) + IPI (3 mg/kg) group had the highest incidence of high-grade AEs, possibly due to the dose-related toxicity of IPI. One study (D'Angelo et al., 2018) supported the finding that a lower dose of IPI (1 mg/kg *vs.* 3 mg/kg) might reduce AE incidence and make this combination therapy safer. Notably, another report (Scherpereel et al., 2019) demonstrated that the safety of NIVO alone or combined with IPI compared favorably with what had been proposed for platinum-based chemotherapy. As the AEs observed in our studies were similar to those reported for immunotherapy drugs used in other settings and in previous trials, we hypothesize that the safety of combination therapy was correlated with drug dose and pretreatment. However, further trials with larger study cohorts are required to validate this hypothesis.

Because the included studies were from different tumors, and because of the dose and sequence of the combination, heterogeneity may also result. However, in clinical practice, advanced tumor progression and outcome vary, but the primary therapeutic goal is to control symptoms and prolong survival, consistent with the results of various studies, and thus heterogeneity may not affect the outcome.

This study had some limitations. First, differences in tumor types may lead to heterogeneity between studies. Second, because of the varying designs of the studies, we could not analyze differences in dosages. Third, we only included phase I/II studies; ongoing studies were not included due to incomplete data.

## Conclusion

In patients with advanced tumors, NIVO + IPI therapy significantly improved ORR, DCR, and mPFS. AEs ≥ grade 3 were more common but were controllable. Due to the limited quality and quantity of the included studies, additional high-quality studies are needed to validate the above conclusions.

## Author Contributions

YY collected and analyzed the data and wrote the article. GJ and YP collected data. PW, YH, and WW prepared the pictures and tables. ZW, HZ, and GT modified the article. ZZ provided the idea. All authors read and approved the final manuscript.

## Funding

This work was supported by the National Natural Science Foundation of China (Nos. 31760259).

## Conflict of Interest

The authors declare that the research was conducted in the absence of any commercial or financial relationships that could be construed as a potential conflict of interest.
